# High prevalence of albuminuria among adult males living with HIV in Botswana

**DOI:** 10.1038/s41598-024-65099-w

**Published:** 2024-06-23

**Authors:** Mosepele Mosepele, Ponego Ponatshego, Kesaobaka Molebatsi, Christopher Williams, Lucky Mokgatlhe, Shahin Lockman, Nabila Youssouf, Robert Gross, Joseph Jarvis, Duolao Wang, Shabbar Jaffar

**Affiliations:** 1 Botswana Harvard Health Partnership, Gaborone, Botswana; 2 Faculty of Medicine, University of Botswana, Gaborone, Botswana; 3 UCL Institute for Global Health, University College London, London, UK; 4 London School of Hygiene and Tropical Medicine, London, UK; 5 Harvard T.H. Chan School of Public Health, Harvard University, Boston, USA; 6 Perelman School of Medicine, University of Pennsylvania, Philadelphia, USA; 7 Department of Statistics, University of Botswana, Gaborone, Botswana; 8 Liverpool School of Tropical Medicine, Liverpool, UK

**Keywords:** Biomarkers, Nephrology, Urinalysis, Diagnosis, Retrovirus

## Abstract

Chronic HIV disease is associated with a fivefold increase in albuminuria outside of sub-Saharan Africa. However, very little is known about albuminuria risk among people living with HIV (PLWH) in sub-Saharan Africa. Therefore, we conducted a cross-sectional observational HIV clinic-based study of albuminuria among 1533 adults aged 21 years or older between January 2020 and January 2021 in Gaborone, Botswana. Clinical albuminuria was defined using a sex-based albumin‒creatinine ratio (ACR) of 25–355 mg/g for females and 17–250 mg/g for males. The study population mean age was 48.5 (SD 10.3) years, and 764/1533 (49.7%) were female. The overall prevalence of albuminuria was 20.7% (95% CI 18.7%, 22.8%). A higher proportion of males were more likely to be categorized as having albuminuria than females, 25% (95% CI 22.0, 28.2) versus 16.4% (95% CI 13.8,19.2), P value < 0.001. In the final multivariate models, predictors of albuminuria differed by sex group. Larger longitudinal studies are required to evaluate the impact of albuminuria among PLWH with particular emphasis on the effect of sex on the risk of albuminuria.

## Introduction

Albuminuria of any degree is associated with excess mortality from major adverse cardiovascular endpoints in the general population^1^. It is assumed that albuminuria may reflect widespread endothelial injury (including in the glomerulus)^2^. The effect of albuminuria on cardiovascular disease (CVD) outcomes has been observed across different populations/races. For instance, in the largest study of albuminuria and cardiac dysfunction among 1815 Hispanic individuals in the USA, microalbuminuria was independently associated with left ventricular hypertrophy and heart failure with preserved ejection fraction^3^, while in a study of 17,753 Chinese adults with self-reported prediabetes and diabetes, persistent proteinuria on a dipstick was independently associated with an increased risk for myocardial infarction (HR 2.23, 95% CI 1.66, 3.01)^4^.

HIV infection is associated with a fivefold increase in the presence of microalbuminuria^5^. An overall loss of greater than or equal to 30 mg/g (albumin to creatinine) loss in urine was associated with 20% 5-year mortality in a cohort of PLWH, while in a cohort of women living with HIV only, any persistent protein in urine (proteinuria) was associated with a fourfold increase in mortality^6,7^. While not directly comparable, these data highlight the importance of studying albuminuria in a high-HIV-prevalence setting such as Botswana.

The prevalence of albuminuria and factors associated with its presence remain unknown among ART-treated black African adults living with HIV, even though detecting albuminuria or microalbuminuria is a relatively non-invasive and technically easy test with more than five licenced point-of-care tests (bedside). The purpose of this study was to determine the prevalence of albuminuria among PLWH in Botswana and to assess predictors of albuminuria using an initial exploratory low-cost prevalence cross-sectional study design. The study enrolled approximately equal numbers of males and females so that we were sufficiently powered to assess whether sex is an effect modifier in the sensitivity analysis, given that in the general population, there are sex-specific cut-off points for clinically significant albuminuria.

## Methods

### Study participants

Between January 2020 and January 2021, we conducted a cross-sectional convenience sampling study to enrol adults aged 21 years or older who were receiving antiretroviral therapy (ART) and virally suppressed in Gaborone, Botswana, during their regular HIV care visits. That is, during each HIV clinic day, screening and enrolment proceeded until the desired number of enrolees was reached. Enrolment took place at the Princess Marina Hospital Infectious Disease Care Clinic (PMH-IDCC) and 4 additional PMH-IDCC satellite clinics. Collectively, PMH-IDCC has approximately 14,000 HIV-infected patients who are currently receiving ART: 7000 at the main clinic and the other 7000 spread across 6 satellite clinics across the city of Gaborone. PMH-IDCC is the oldest and largest HIV clinic in the country. The study protocol, informed consent, and other materials were reviewed and approved by the ethics boards of the University of Botswana and the Health Research and Development Committee (IRB of the Botswana Ministry of Health and Wellness). All study participants provided written informed consent prior to study procedures. All study procedures were performed according to the IRB approval in accordance with the principles the Declaration of Helsinki.

### Study procedures

Patients were recruited when they attended the clinic for routine HIV care. Recruitment aimed to achieve balance by sex group to allow for robust analysis by sex group. In keeping with clinical practice, patients were told about the study during the daily morning patient information sessions. Following that, members of the study team approached patients at a time and place that was convenient and provided adequate privacy within the clinic building for consenting and enrolment. Patients were excluded from participating if they reported any factor associated with albuminuria, such as fever, strenuous exercise, severe muscle injury/inflammation, recent use of medications that can cause muscle injury, etc.

Participants underwent an initial screening interview to verify the sources of all medical information. Their medical records were reviewed, and all relevant medical information was abstracted. Information on HIV parameters (viral loads, CD4 counts, ART history, etc.), general medical comorbidities (hypertension, DM II, CKD, etc.) and medication list was abstracted from the medical records. Multimorbidity was defined as having any two or more of any of the following conditions: diabetes mellitus, chronic kidney diseases, hypertension, malignancy, or other chronic medical condition.

### Laboratory testing

All participants provided a one-time, random urine sample using the study collection bottles. Urinary creatinine was measured using a colorimetric assay (Jaffe method)^8^. Urinary albumin was measured using an immunoturbidimetric assay. The tests were run on an existing Cobas Integra© machine at the Botswana-Harvard Health Partnership Reference Research Laboratory (BHRRL), Gaborone, Botswana. This laboratory is situated on PMH grounds, which is the same location as the main HIV clinic. The remaining urine samples were stored for future analysis.

### Albuminuria assessment

Albuminuria was defined using the sex-based albumin‒creatinine ratio (ACR). Albuminuria was defined as 25–355 mg/g for females versus 17–250 mg/g for males^9^.

### Statistical analysis

All continuous baseline characteristics were assessed for normality visually and using the Shapiro‒Wilk test^10^. In our preliminary analysis, the distributions of all available continuous characteristics were skewed. Therefore, a Mann‒Whitney *U* test was used to compare those variables between participants with and without albuminuria. For categorical characteristics, we used a Chi-square test and Fisher’s exact test whenever we obtained expected counts that were less than 5^11^.

To identify clinical predictors of albuminuria in the entire cross-sectional study, we first fitted univariable modified Poisson regression models with robust error variance. All predictors that had P values of 0.2 or less in the univariable model were included in the multivariable model.

Prior to final model building we considered the possible effects of effect modification and collinearity.

First, because albuminuria can vary by sex as discussed above, we assessed for effect modification by sex by including an interaction term of sex and all predictors with a P-value less than 0.2 in the univariable model (presented in supplementary material). If interaction analysis reveals effect modification, we will present multivariate modified Poisson regression results stratified for men and women separately. However, clinical factors were first assessed for collinearity using Spearman’s correlation coefficient before selecting them for inclusion in the multivariate model^12,13^. If two variables were found to have a statistically significant correlation coefficient, only one of them was included in the multivariate model. In sensitivity analysis, we exchanged collinear variables in the final multivariate model to assess whether the direction and magnitude of the association remained the same. We reported adjusted risk ratios (aRR), corresponding 95% confidence intervals and P values for all these models. P values less than 0.05 indicated significant differences or associations.

## Results

### Baseline characteristics

Of the 1537 enrolled participants, 1533 participants had complete data to be included in this analysis. They were approximately sex balanced, with 764 (49.7%) females and a mean age of 48.5 (SD 10.3) years, and the prevalence of albuminuria was 20.7% (95% CI 18.7%, 22.8%). Among the 318 participants with albuminuria, the proportion of men with albuminuria was higher than that of women [(25.0% vs. 16.4%, P < 0.001]. Additional relative distributions of baseline factors according to albuminuria group are described in Table 1.

**Table 1:** Means (standard deviations), frequencies (percentages) and corresponding P values for baseline characteristics of study participants stratified by albuminuria status.

Demographic	Overall (n = 1533)	Non-Albuminuria group (n = 1215, 79.3%)	Albuminuria group (n = 318, 20.7%)	P value
Age^2^, years (SD)	48.5 (10.3)	47.8 (10.2)	51 (10.3)	< 0.001
Females, N (%)	764 (49.7)	638 (52.5)	125 (39.3)	< 0.001
Males	773 (50.3)	577 (47.5)	193 (60.7)	
Waist–hip ratio (Female ≥ 0.85 cm, Male ≥ 0.90 cm)	790 (51.4)	591 (48.6)	196 (61.6)	< 0.001
Diabetes mellitus	31 (2)	20 (1.6)	11 (3.5)	0.069
Hypertension	451 (29.3)	328 (27)	121 (38.1)	< 0.001
Chronic kidney disease^1^	2 (0.1)	1 (0.1)	1 (0.3)	0.372
Malignancy^1^	9 (0.6)	9 (0.7)	0	0.218
Multimorbidity*	28 (1.8)	18 (1.5)	10 (3.1)	0.083
Systolic BP^2^ (mmHg)	126 (18.9)	125 (17.9)	131 (21.8)	< 0.001
Diastolic BP^2^ (mmHg)	82.7 (12.2)	82 (11.7)	85.5 (13.7)	< 0.001
HIV duration^a^ (years)	14.3 (9.6–16.8)	14.2 (9.3–16.7)	14.4 (9.8–17.0)	0.939
ART duration^a^ (years)	13.4 (8.5–16.0)	13.1 (8.1–15.8)	13.7 (9.1–16.2)	0.249

Superscript^1^ implies that the P value for Fisher’s exact test is reported. Superscript^2^ implies that the mean, standard deviation and P value for the Mann‒Whitney U test are reported. Superscript^a^ implies that median, interquartile range and P value for Mood’s test of medians are reported. *Having any two or more of the following: diabetes mellitus, hypertension, chronic kidney disease and malignancy. *BP* blood pressure, *HIV* human immunodeficiency syndrome, *ART* antiretroviral therapy.

### Univariable models for the entire cohort, males and females separately

Table 2 shows estimated risk ratios from the univariable analysis for predictors of albuminuria in the entire cohort and then separately for males and females. Overall, there were more clinical predictors of albuminuria for males than females (Table 2).

**Table 2:** Unadjusted risk ratios (RR), 95% confidence intervals (CI) and corresponding P values of clinical predictors of albuminuria for all participants, males only participants and females only participants.

Clinical factors	All participants		Males		Females	
	RR (95% CI)	P value	RR (95% CI)	P value	RR (95% CI)	P value
Age (per 5-year increase)	1.12 (1.07, 1.18)	< 0.001	1.14 (1.08, 1.20)	< 0.001	1.07 (0.99, 1.16)	0.093
Males	1.53 (1.25, 1.87)	< 0.001				
Waist–hip ratio (Female ≥ 0.85, Male ≥ 0.90)	1.49 (1.22, 1.83)	< 0.001	1.61 (1.25, 2.07)	< 0.001	1.46 (1.04, 2.06)	0.030
Diabetes mellitus	1.76 (1.08, 2.85)	0.023	1.63 (0.87, 3.07)	0.129	1.97 (0.93, 4.14)	0.075
Hypertension	1.47 (1.21, 1.78)	< 0.001	1.90 (1.49, 2.42)	< 0.001	1.23 (0.88, 1.70)	0.224
Chronic kidney disease^nec^	2.44 (0.61, 9.80)	0.210	4.05 (3.58, 4.58)	< 0.001	–	–
Cancer Malignancy^nec^	–	–	–	–	–	–
Multi-morbidity*	1.75 (1.05, 2.90)	0.031	2.21 (1.27, 3.85)	0.005	1.45 (0.61, 3.47)	0.403
Systolic blood pressure^mc^ (per 2-mmHg increment)	1.03 (1.02, 1.04)	< 0.001	1.03 (1.02, 1.04)	< 0.001	1.02 (1.00, 1.04)	0.045
Diastolic blood pressure (per 2-mmHg increment)^mc^	1.04 (1.02, 1.05)	< 0.001	1.04 (1.02, 1.06)	< 0.001	1.03 (1.00, 1.05)	0.076
HIV disease duration (per 3-year increase)^mc^	1.02 (0.96, 1.07)	0.570	1.07 (1.01, 1.15)	0.028	1.00 (0.91, 1.11)	0.954
Duration on ART^mc^ (per 3-year increase)	1.05 (0.99, 1.12)	0.089	1.11 (1.03, 1.19)	0.007	1.05 (0.94, 1.17)	0.374

Superscript ^mc^ implies that the corresponding variable was removed in the adjusted model to prevent multicollinearity; ^nec^ implies that there were not enough cases to calculate risk ratios (RRs). *Having any two or more of the following: diabetes mellitus, hypertension, chronic kidney disease and malignancy. *HIV* human immunodeficiency syndrome, *ART* antiretroviral therapy.

### Stratified multivariate models for males and females

Multicollinearity was identified among the following pairs of variables: HIV and ART duration (r = 0.90, P < 0.001), systolic and diastolic blood pressure (r = 0.79, P < 0.01) but not diastolic blood pressure/systolic blood pressure and hypertension (r = 0.3, P < 0.001). As shown in Table 3, while only age was associated with albuminuria for both males and females, aRR 1.08 [(95% CI 1.00, 1.15) P = 0.038] and aRR 0.04 [(95% CI 0.01, 0.150, P < 0.001), respectively, no other clinical predictor was associated with albuminuria among females. Supplementary Table 2a shows that clinical predictors of albuminuria among men (Table 3) were similar for the entire cohort (except for hypertension). In the sensitivity analysis, we reran the final models with multimorbidity (instead of Diabetes Mellitus) and systolic blood pressure (instead of diastolic duration), and our associations remained similar although weaker when compared to prior models (Supplementary Table 2b).

**Table 3:** Adjusted risk ratios (RR), 95% confidence intervals (CI) and corresponding P values of clinical predictors of albuminuria stratified by sex.

Clinical factors	Male		Female	
	aRR (95% CI)	P value	aRR (95% CI)	P value
Age (per 5-year increase)	1.08 (1.00, 1.15)	0.038	0.04 (0.01, 0.15)	< 0.001
Waist–hip ratio (Female ≥ 0.85, Male ≥ 0.90)	1.34 (1.03, 1.74)	0.029	1.35 (0.95, 1.93)	0.099
Diabetes Mellitus	1.23 (0.65, 2.34)	0.528	1.77 (0.82, 3.81)	0.144
Hypertension^mc^	1.34 (1.02,1.77)	0.039	n/a	n/a
Diastolic Blood pressure^mc^ (per 2-mmHg increment)	1.03 (1.01, 1.05)	0.012	1.02 (0.99, 1.05)	0.159
Duration on ART (per 3-year increase)	1.08 (0.70, 1.67)	0.725	n/a	n/a

Superscript ^mc^ implies that the corresponding variable was removed in the adjusted model to prevent multicollinearity; *ART* antiretroviral therapy.

## Discussion

The study shows a high prevalence of albuminuria in a cohort of people living with HIV (PLWH) in Botswana in sub-Saharan Africa (SSA). Approximately one in five of the participants had albuminuria. This estimate is comparable to findings in similar settings. In a smaller cohort of 903 rural South African PLWH, Wensink et al. found that albuminuria was present in 20% of patients^14^. In a Ugandan study of albuminuria conducted in ART-naïve patients, the albuminuria prevalence was 18.9% in that population^15^. This prevalence of albuminuria in our larger cohort of PLWH therefore confirms what has been observed elsewhere and raises serious concerns about the possibility of future end-organ dysfunction, such as cardiovascular events, among ageing HIV cohorts in SSA^16–19^.

Albuminuria was more common in males (25.0%) than in females (16.4%). Few recent data on albuminuria overall or by sex group among ART-treated patients in SSA are available for comparison^20–25^. Our study critically highlights a clear sex-linked difference in the prevalence of albuminuria. In some non-HIV cohorts, it has been noted that females have lower albuminuria, better albuminuria responses and overall decreased rates of low glomerular filtration rate (GFR), with some studies attributing this difference to the possibility of better compliance with healthy lifestyles rather than just anthropometric measurements by sex^26^. More data are needed in this setting to further assess this difference and any additional contributing factors. Unfortunately, the two studies of albuminuria performed in South Africa (N = 903) and Uganda (N = 185) enrolled fewer men (< 40% of enrolees); hence, these low numbers may have precluded the ability to detect the effect of sex on albuminuria in those cohorts^14,15^. However, given that at least 1 in 4 men living with HIV had albuminuria in this Botswanan cohort is a significant finding that will need follow-up study.

As there are suggestions in the literature that there are variations regarding sex-specific ACR cut-off points that are used to classify participants as having albuminuria or not, we accounted for effect modification by sex in our analysis. Under the stratified analysis, age was statistically significantly associated with albuminuria among both males and females. Among males only, risk factors for albuminuria were elevated waist–hip ratio, having hypertension, plus higher diastolic blood pressure. This confirms the same clinical risk factors for albuminuria that have been observed in both HIV and non-HIV cohorts. Future studies should further assess whether these marked sex differences are true and establish sex-specific mediating factors by HIV group and then other common comorbidities among PLWH.

Predictors of albuminuria differ among males and females and may also be driven by underlying chronic medical conditions^27^. Several studies have shown that PLWH with diabetes mellitus have a significantly higher prevalence of albuminuria^28–30^. For instance, HIV and diabetes mellitus comorbidity was associated with a twofold greater risk of albuminuria when compared to individuals with either disease alone^31^. While we also reported that diabetes mellitus was associated with albuminuria among PLWH only in unadjusted analysis, we had very few PLWH with comorbid diabetes mellitus (N = 11); hence, we could not fully study the effect of this comorbidity on the outcome of albuminuria.

Several studies have shown that generally, many patients with albuminuria experienced improvements in their albuminuria following ART initiation, although the same degree of improvement was not experienced with initiating TDF/FTC in several of the studies^32,33^. Few data exist on albuminuria trends with long-term ART exposure. Our study has demonstrated that longer ART duration is significantly associated with albuminuria. We report this with caution, as the duration of HIV vs. ART exposure may merely reflect ageing, since age was a predictor of albuminuria in our models. Therefore, the roles of both HIV disease and ART (and ART regimen) in the development of albuminuria need to be further studied.

There are limited data on the association of albuminuria and isolated blood pressure among PLWH. A study conducted in a Chinese population concluded that there is a J-shaped phenomenon when investigating single and combined effects of SBP and DBP on albuminuria. They observed that the risk of albuminuria was significantly associated with DBP in participants with an SBP of at least 130 mmHg and with SBP in participants with a DBP of at least 80 mmHg and inversely significantly associated with SBP in participants with a DBP below 70 mmHg^34–36^. This complex relationship between albuminuria and blood pressure requires further investigation, as it might guide the definition of appropriate blood pressure targets/cut-off points (for both or either systolic versus diastolic blood pressure) among PLHW with albuminuria. This is particularly important because blood pressure was associated with albuminuria in our study.

There were limitations to our study; the study design involved a convenience sample rather than a random sample. Patients were approached as they came for their regular ART visit to determine their eligibility and then consent and enrol if they agreed to participate in the study; hence, there is a possibility of selection bias. A single measurement of urine albumin excretion could result in misleading classifications of albuminuria. However, repeated measurement is both time-consuming and more costly and is therefore not feasible as a nationwide screening tool for PLWH who are traditionally at a higher risk for albuminuria. We did not assess the effect of ACEI/ARB exposure on the risk of albuminuria, even though ACEIs and ARBs are well known to influence the degree of albuminuria. We believe that a longitudinal study is best suited to explore this association. There is possible confounding from unmeasured variables such as bodyweight and other characteristics that we did not collect. However, future randomized controlled trials could be used to address this problem. With regard to the higher prevalence of albuminuria among men, our results may be biased because we did not assess the effect of sex-specific roles such as actual physical work that either men or women undertook around the time of enrolment. Future studies should collect data on actual physical work performed by both males and females in studies of albuminuria because men are more likely to perform manual work than women in settings such as Botswana. Finally, this study did not establish a causal relationship because of its cross-sectional nature. Thus, the possibilities of causal relationships remain to be elucidated by prospective observation of the relationships between risk factors outlined in this study and the development of albuminuria.

In conclusion, albuminuria prevalence was high in this cohort of PLWH in Botswana, with approximately one in five of the participants overall and one in four (25%) males being categorized as having albuminuria during a one-time assessment in an HIV clinic. This is significant, as albuminuria increases the probability of developing cardiovascular and renal diseases. Notably, predictors of albuminuria differed by sex group. Among males, modifiable traditional risk factors for albuminuria, such as elevated diastolic blood pressure and waist–hip ratio, remained important predictors of albuminuria in a cohort of adult men living with HIV. The effect of chronic ART vs. HIV disease duration remains poorly understood but urgently requires further evaluation, as chronic ART exposure was associated with a twofold increase in the risk for albuminuria.

### Supplementary Information

Supplementary Tables.

## Data Availability

All the data supporting the findings are available and will be shared as per the rules of the ethics committees that approved this study. Any parties requesting data from this study should contact both the corresponding author (Mosepele Mosepele) and Ministry of Health of Botswana Human Research Subjects Ethics Committee at (hhealthresearch@govbots.onmicrosoft.com).
