# Supplementary material for: High prevalence of albuminuria among adult males living with HIV in Botswana
Source: Sci Rep. 2024 Jun 23;14:14432. doi: 10.1038/s41598-024-65099-w (PMC11194263; doi:10.1038/s41598-024-65099-w)
Supplement: Supplementary file 1 — Supplementary Tables. [file 41598_2024_65099_MOESM1_ESM.pdf]

Title: High Prevalence of Albuminuria among Adult Males Living with HIV in Botswana

Authors: Mosepele Mosepele, Ponego Ponatshego, Kesaobaka Molebatsi, Christopher Williams, Lucky Mokgatlhe, Shahin Lockman, Nabila Youssouf, Robert Gross, Joseph Jarvis, Duolao Wang & Shabbar Jaffar

Supplementary Table 1. Risk ratios (RR) and interaction effects, 95% confidence intervals (CI) and corresponding p-values between each clinical or demographic factors and sex.

Clinical Factors	Risk Ratio (95% CI)	P value
Sex (Male)	0.83 (0.31, 2.21)	0.714
Age (per 5-year increase)	1.08 (1.00, 1.16)	0.060
Age*Sex (Male)	1.06 (0.96, 1.16)	0.232
Sex (Male)	1.49 (1.05, 2.09)	0.024
Waist-hip ratio (Female≥0.85, Male≥0.90)	1.49 (1.06, 2.09)	0.022
WHR*Sex (Male)	1.12 (0.73, 1.71)	0.609
Sex (Male)	1.54 (1.26, 1.89)	<0.001
Diabetes	1.95 (0.92, 4.10)	0.080
Diabetes*Sex (Male)	0.83 (0.31, 2.21)	0.709
Sex (Male)	1.37 (1.05, 1.78)	0.019
Hypertension	1.23 (0.89, 1.71)	0.210
Hypertension*Sex (Male)	1.54 (1.03, 2.31)	**0.037**
Sex (Male)	1.52 (1.24, 1.87)	<0.001
Multi-morbidity*	1.45 (0.61, 3.47)	0.403
Multi-morbidity* *Sex (Male)	1.53 (0.54, 4.29)	0.423
Sex (Male)	0.78 (0.20, 3.08)	0.727
Systolic Blood pressure (per 2-mmHg increment)	1.02 (1.00, 1.04)	0.063
SBP*Sex (Male)	1.01 (0.99, (1.03)	0.375
Sex (Male)	0.87 (0.21, 3.58)	0.842
Diastolic Blood pressure (per 2-mmHg increment)	1.03 (1.00, 1.06)	0.071
DBP*Sex (Male)	1.01 (0.98, 1.05)	0.433
Sex (Male)	1.31 (0.74, 2.32)	0.355
Duration on ART (per 3-year increase)	1.20 (0.54, 2.68)	0.660
Duration on ART*Sex (Male)	1.39 (0.57, 3.43)	0.472

*Having any two or more of the following: diabetes mellitus, hypertension, chronic kidney disease and malignancy: WHR; Wait hip ratio: SBP; Systolic blood pressure: DBP; Diastolic blood pressure: ART; Antiretroviral therapy

Supplementary Table 2a. Adjusted risk ratio (aRR), 95% confidence intervals and corresponding p-value of modified Poisson regression model of albuminuria

Clinical factors	aRR	95% CI	p-Value
Age (per 5-year increase)	1.07	1.01 – 1.13	0.019
Sex (Male)	1.35	1.03 – 1.77	0.029
Waist-hip Ratio	1.34	1.09 – 1.66	0.007
Diabetes	1.44	0.88 – 2.36	0.153
Hypertension	0.94	0.67 – 1.33	0.735
Diastolic Blood pressure (per 2-mmHg increment)	1.02	1.01 – 1.04	0.006
Duration on ART (per 3-year increase, log transformed)	1.04	0.71 – 1.51	0.860
Hypertension*Sex (Male)	1.44	0.96 – 2.18	0.081

ART; Antiretroviral therapy

Supplementary Table 2b. Sensitivity analysis of the main model (shown in supplementary Table 2a)

Clinical factors	aRR	95% CI	p-Value
Age (per 5-year increase)	1.06	1.00 – 1.12	0.048
Sex (Male)	1.31	1.00 – 1.72	0.050
Waist-hip Ratio	1.33	1.08 – 1.65	0.008
Multi-morbidity*	1.40	0.83 – 2.35	0.204
Hypertension	0.92	0.65 – 1.31	0.648
Systolic Blood pressure (per 2-mmHg increment)**	1.02	1.01 – 1.03	0.001
Duration on ART (per 3-year increase, log10 transformed)	1.08	0.74 – 1.59	0.694
Hypertension*Sex (Male)	1.48	0.98 – 2.24	0.060

*Replaced diabetes from the main model
**Replaced diastolic blood pressure from the main model
ART; Antiretroviral therapy
